# Efficacy of Protein Baits with Fipronil to Control *Vespa velutina nigrithorax* (Lepeletier, 1836) in Apiaries

**DOI:** 10.3390/ani13132075

**Published:** 2023-06-23

**Authors:** Jesús F. Barandika, Omaira de la Hera, Roberto Fañanás, Arrate Rivas, Eugenia Arroyo, Rosa M. Alonso, M. Luz Alonso, Egoitz Galartza, Aitor Cevidanes, Ana L. García-Pérez

**Affiliations:** 1 NEIKER-Basque Institute for Agricultural Research and Development, Basque Research and Technology Alliance (BRTA), 48160 Derio, Spain; 2 Zientzia eta Teknologia Facultatea, Euskal Herriko Universitatea, 48940 Leioa, Spain; 3 D+S-OABE, Poligono Industrial Zabale, Parcela III, 48410 Orozko, Spain; 4 Regueta Kalea 1, 31241 Arbeiza, Spain

**Keywords:** *Vespa velutina*, protein baits, fipronil, control, beekeeping

## Abstract

**Simple Summary:**

Since the accidental introduction of the yellow-legged hornet (Vespa velutina nigrithorax) at the beginning of 21st century in Europe, it has become a threat to many pollinators, including domestic bees. After its arrival in France in 2004, it quickly spread across the continent, reaching Gipuzkoa (Basque Country) in 2010, where it poses a serious problem for beekeeping. To reduce this problem, various control strategies have been developed, such as the removal of nests or the capture of founder queens in spring. However, these methods have not been effective in reducing the impact of hornets on beekeeping. The use of protein baits with biocides has shown to be an effective method to control invasive wasp populations in natural environments, however, they have not been used to control V. velutina. This study evaluated the efficacy of these baits in reducing the impact of hornets in apiaries. The results have shown that when the presence of hornets in apiaries is high, high-bait consumption is produced, leading to a significant reduction in the number of hornets within 48 h. This reduction lasts for at least two weeks after baiting and allows the honey bees to recover and return to their normal activity.

**Abstract:**

The yellow-legged hornet (*Vespa velutina nigrithorax*), outside its natural range, has become a major threat to domestic bees. Several control methods have been used to fight against *V. velutina*, but the results achieved are not satisfactory. The use of protein baits with biocides has shown to be an effective method to control invasive wasp populations, but they have not been used to control *V. velutina*. Thus, the efficacy of protein baits containing fipronil to reduce the presence of hornets in apiaries was evaluated in this study. After laboratory determination of the optimal efficacy of a protein bait at a 0.01% concentration of fipronil, field trials were conducted involving 222 beekeepers. The data reported by the 90 beekeepers who completed the requested questionnaire demonstrated that in the groups of apiaries with the highest pressure of hornets (groups with 10–30 and >30 hornets), there was a significant decrease in the presence of *V. velutina*, lasting at least two weeks. The reduction in the number of hornets was positively correlated with bait consumption, and bait consumption was positively correlated with the number of hornets present at the time of treatment. Although the method used has shown good efficacy and the concentration of fipronil used was very low; possible negative effects on the environment should also be evaluated.

## 1. Introduction

The interconnection of world economies, especially trade, has generated a movement of people, goods and technologies, which has accidentally led to an increase in the number of alien species [1]. Some of these species adapt to the new environmental system and may even have a negative impact on the ecosystem [2]. In Europe, there are thousands of invasive species, a large part of which are considered pests due to the interference or harm they produce to other populations [3]. Several insects, including social insects such as termites, wasps, ants and hornets, are on the list of the most damaging invasive species in the world, according to the International Union for Conservation of Nature (IUCN) [1,4].

Regarding hornets, not all invasions establish successfully. However, one of the recent exceptions is *Vespa velutina nigrithorax* (*V. velutina*), also called yellow-legged hornet or Asian hornet, which was accidentally introduced in France in 2004 [5,6]. This specie has spread in different countries in Europe, such as Italy, Belgium, the United Kingdom, Spain, Germany, and Portugal [7], and recently, it has also been reported in Ireland [8] and Switzerland [9]. Therefore, it is the first successful invasion of the Vespidae family on this continent [10].

*Vespa velutina* is a eusocial hymenopteran that lives most of its vital cycle in colonies that are composed of thousands of individuals [11,12]. The life cycle of *V. velutina* is similar to that of other social wasps or hornets [13]. The cycle starts in spring, when the new founder queens start the construction of the primary nest, which is found in places protected from the weather and predators. First, it is composed of a small number of cells where the queen lays the first eggs. Once the workers are born, they will contribute to the development of the colony, whereas the queen stays in the nest laying eggs. The colony is still growing until mid-summer when it reaches its largest size. If there is not enough space to grow, hornets move in search of larger spaces to construct secondary nests and often find them in trees and, in general, high in trees but also in houses, under roofs, on balconies, in empty buildings, etc., and in recent years, nests have also been found at ground level [11]. In autumn, when the nest reaches maturity, the mating period starts. The low temperatures and the lack of food produce the death of the founder queen and the nest. At this time, the fertilized and overfed future queens (gynes) leave the nest for sheltered places, where they spend their lethargy to endure the winter and start a new colony in spring [11,14,15].

Because of the need for protein to feed the larvae and the need for carbohydrates for the adult hornets, mid-summer and early autumn are the periods when hornets massively attack apiaries and crops [16,17,18,19]. Therefore, crops are lost, honey production and the number of pollinating insects are reduced, involving a large economic loss in the agricultural and beekeeping sector and a serious biodiversity problem [19]. Furthermore, *V. velutina* is also a threat to public health. Their bite can cause serious health problems, especially for allergic people [20,21]. Although cases of attack on humans are rare, they can occur if hornets feel threatened or if people get too close to their nests [22].

The damage caused by *V. velutina* in beekeeping has triggered the development of different control methods, both biological and physical, to reduce its spread and minimize its economic impact [23]. Biological control methods may include the use of parasitic insects, viruses or fungi that infect larvae and adult hornets, helping to reduce their population. Unfortunately, these studies are limited, and the results are still inconclusive [24,25]. Meanwhile, the main physical methods are based on the detection and removal of nests [26], the use of baited traps to capture founder queens and workers [27,28,29], or beehive muzzles [30], among others.

Commercial or homemade traps consist mainly of sugary or protein substances attractive to social vespids with an alcoholic component added as a repellent for honey bees [28,31,32,33]. These attractive traps are placed surrounding the apiary to reduce the pressure of hornets. However, their overuse is not recommended due to their low specificity for *V. velutina* and because of their impact on nontarget species [33]. Other methods, such as electric harps, consist of a frame with parallel electrified wires that produce an electric shock when touched by a flying insect. Preliminary results of their use complemented with other methods contribute to reducing the impact of *V. velutina* on apiaries [32]. Beehive muzzles [30] are a biodiversity-friendly method that reduces foraging paralysis and likely enhances the survivorship of colonies, but it does not kill the hornets. In addition, there are other types of traps currently in progress based on attractive pheromones, whose main objective is to trap the males, thus reducing the fecundation of the gynes [34,35,36].

Other methods to control Vespidae, such as Vespula germanica, consist of the use of protein baits containing biocides [25,37,38,39,40]. Protein baits with biocides are especially interesting in the beekeeping sector to reduce hornet attacks on apiaries. Baits are placed near the entrance of the hives with the objective of the hornets picking them up and introducing them into the nest to feed the larvae. The biocide decreases nest activity by killing the larvae [41]. According to the literature, biocidal baits are based on fish, meat, synthetic proteins, or paraffin wax [42,43,44]. The biocide is added to the different matrices free or encapsulated [45].

Most of the protein baits commercialized for the control of the Vespidae family contain fipronil as the biocide in their composition [41,42,46,47]. Fipronil is a systemic insecticide of the phenyl pyrazole family that acts by blocking neuronal inhibitory receptors, leading to neuronal hyperexcitability produced by the accumulation of the neurotransmitter GABA in the synaptic cleft, generating the death of the individual [48]. The use of active fipronil was approved by the European Union N° 540/2011 Implementing Regulation of the 25 of May of 2011 Commission [49]. However, since it is not selective for *V. velutina*, it is important to use the minimum dose to inactivate a nest, killing larvae and adults, since eggs and sealed brood stages would not be affected by the baits.

The aims of this work were (i) to establish the optimal concentration of fipronil in the protein bait to kill the larvae of *V. velutina*. For that purpose, groups of larvae were fed in the laboratory with protein baits containing different concentrations of the biocide. The concentration of fipronil in the dead larvae was determined by a validated high-performance liquid chromatography-diode array detector (HPLC-DAD) analytical method. Before the analysis, QuEChERS (Quick, Easy, Cheap, Effective, Rugged and Safe) dispersive solid phase extraction methodology was applied for sample treatment; (ii) to study the efficacy of the protein baits with fipronil in minimizing the presence of *V. velutina* in apiaries in field trials carried out from 2019–2021.

## 2. Materials and Methods

### 2.1. Larvae Collection

Secondary nests were collected by trained personnel and supplied in transparent plastic boxes with holes in the lid to allow the hornets to breathe. To extract the combs with the larvae from the nests, the hornets were anesthetized using diethyl ether (99.7%) (Panreac Applichem, Barcelona, Spain). The anesthetic was added progressively until all the hornets were asleep. The nests were examined, the hornets were removed, and the combs that contained larvae were separated and placed individually in transparent plastic boxes. Then, the larvae in each comb were counted.

### 2.2. Larval Inactivation Assays in the Laboratory

A blank protein bait was supplied by D+S-OABE, S.L. (Orozko, Biscay, Spain). From this blank protein bait, five bait mashes were prepared to facilitate the feeding of the larvae and simulate their current feed process by the hornets. One consisted of a blank bait mash without biocide to monitor the normal activity of the larvae. The remaining four contained different concentrations [0.003, 0.004, 0.007 and 0.01% (*w*/*w*)] of fipronil (Fipronil technical, purity: 95.4%). For the preparation of the bait mashes, 6 g of protein blank bait was weighed, and 15 mL of Milli-Q ultra-pure water (18.2 MΩcm) (Milli-Q Advantage A10 System, Merck Millipore, Darmstadt, Germany) was added. The mixture was homogenized until a mash consistency was obtained, fipronil concentrations were added, and the mixtures were homogenized and stored in the freezer until use.

Combs containing between 25 and 50 larvae were placed in different plastic containers. Larvae from each comb were fed with a different bait. One drop of bait (0.0116 *±* 0.0008 g) was supplied to each larva with the help of a Pasteur pipette. Then, the activity of the larvae was observed periodically. The response to the stimuli, such as approaching the pipette tip close to the larva’s mouth, was taken as a sign of normal activity. Inactive larvae were defined as those that did not respond to any stimuli, neither to touch nor when food was offered, but only showed abdominal movement. After 24 h, the number of inactive larvae in each comb not responding to any food stimulus was counted. The time taken for all larvae in the combs to die was registered. Once the larvae died, they were then frozen (−80 °C) until analysis by HPLC-DAD.

Newly hatched hornets during the assays were removed to work safely. It is important to note that larvae in the sealed brood process did not react to feeding and continued with pupa formation. Therefore, they were not considered in the study.

### 2.3. Analysis of Fipronil Content in Dead Larvae

Larvae stages are complex matrices and need a previous treatment procedure before analyses. QuEChERS (Agilent Technologies, Santa Clara, CA, USA), a dispersive solid phase extraction, was selected for sample treatment. This consists of two steps: extraction of the compound and clean-up of the sample. Additionally, a bait containing 0.01% *w*/*w* fipronil, supplied by the chemical company, was used as a quality control. The dispersive solid phase extraction procedure was applied to this matrix before injection into the chromatographic system.

Six pools of seven dead larvae fed with a bait mash containing 0.01% fipronil belonging to one comb were analyzed. Each pool of larvae, weighing 1–2 g (mean larval weight: 202 mg), was placed in 2 mL vials. Larvae were homogenized using a Tissuelyser Precellys 24 homogenizer (Bertin Instruments, Montigny-le-Bretonneux, France) with seven 3 mm diameter zirconium balls for 3 cycles of 20 s at 4000 rpm and waiting for 30 *s* between cycles. Then, 9 mL of acetonitrile (ACN) was added to the homogenate, and the mix was subjected to the same procedure of the QuEChERS method as the bait up to the frozen step. Before HPLC-DAD analysis, the obtained supernatant was filtered (0.45 µm) and evaporated to dryness using a TurvoVap*^®^* evaporator (Zymark, Hopkinton, MA, USA) with a nitrogen flow at 50 °C. The residue was reconstituted in 250 µL of ACN and filtered again (0.45 µm).

One gram of bait containing 0.01% fipronil was weighed, and 9 mL of ACN was added to a Falcon tube. The mixture was homogenized (2 min, 28,000 rpm) with an Ultraturrax T25 dispersive homogenizer (IKA, Staufen, Germany), and 650 mg of the components of the first extraction step of the QuEChERS method were added to the homogenized sample, shaken manually (1 min) and centrifuged (15 min, 8000 rpm, 15 °C). Then, the reagents of the clean-up step of QuEChERS were added to the supernatant to remove the remaining water, organic acids, fatty acids and sugar from the sample. The same procedure followed in the first extraction step was applied. The obtained supernatant was frozen at −20 °C overnight to precipitate the remaining lipids. Prior to HPLC-DAD analysis, the sample was filtered using a 0.45 µm filter.

The liquid chromatographic system consisted of a Waters 2695 separation module coupled to a Waters 996 diode-array detector (HPLC-DAD) (Waters, Milford, MA, USA). It was used for the determination of fipronil content in the larvae and baits used in the field trials.

The chromatographic separation was carried out in isocratic elution mode on an ABZ+Plus C18 column (25 cm × 4.6 mm, 5 µm) in combination with a precolumn C18 (4 *×* 3 mm) (Sigma Aldrich, St. Louis, MO, USA). The temperature of the column was set at 40 °C, and the samples were kept in the autosampler at 4 °C. The mobile phase used was water acetonitrile (35:65% *v*/*v*) at a flow rate of 1 mL/min. The injection volume was 10 µL. The working wavelength used was 277 nm, corresponding to the maximum of the absorption spectrum of fipronil. The analytical method developed was validated according to the SANTE guide of the European Union for the analysis of pesticide residues in food [50].

### 2.4. Study Area and Selection of Beekeepers

The study of the efficacy of protein baits to minimize the presence of *V. velutina* in apiaries was carried out in the province of Gipuzkoa (Basque Country). The climate in this province is Atlantic, with mild temperatures and abundant rainfall throughout the year. Beekeeping in Gipuzkoa is mainly a recreational activity, with a few professional beekeepers. In fact, although there are many registered apiaries (*n* = 533), the number of hives is not high (*n* = 5336). Thus, considering the beekeeping censuses from 2011 to 2020, 74% of beekeepers owned between 1 and 10 hives, and the density in the province was 2.8 hives/km^2^.

The study was carried out in collaboration with the Gipuzkoa Beekeepers Association (GBA), which has approximately 500 members. To select the participating beekeepers, the GBA contacted its members to inform them of the conditions under which the tests should be carried out and whether they were willing to comply with them.

### 2.5. Testing the Efficacy of Baits in Apiaries

To check the efficacy of the protein baits, field work took place between August and October in 2019, 2020 and 2021. Official authorization was obtained from the Spanish Ministry of Agriculture, Food and Environment (Ref ES-0015049-0000) and the Provincial Council of Gipuzkoa (Ref. 3 May 2019/n° 3330).

Four types of protein baits were used, three made with fish and one with meat. All had the same concentration of fipronil (0.01% *w*/*w*). As mentioned above, the baits were prepared by DTS-Oabe, S.L. Between 50 and 100 g of bait was packaged in small recyclable containers and kept frozen (−20 °C) until utilization in the apiaries. The distribution of the baits to the beekeepers was carried out at the GBA headquarters, where technicians took note of the quantity of bait provided to each beekeeper, the number of apiaries and hives to be treated and the locality where they were placed.

To perform the trials, beekeepers who agreed to participate were informed about a number of prerequisites to be met, (i) a minimum number of hornets should be present in the apiary (>2 hornets in front of each hive), (ii) days when rain and/or strong winds should be avoided, (iii) on the day of treatment, the entrance of the hives should be closed before dawn and kept closed for 4 h, a period in which baits were placed near the hives.

Thus, one hour after sunrise, once the baits had thawed and tempered, a small container with approximately 50 g of bait for every 4–5 hives was placed next to the hive entrance. As the bait was consumed, more bait was added. At the end of the day, the containers were collected and disposed of at a clean point.

To determine the effectiveness of the protein baits, beekeepers had to complete a small questionnaire taking note of the amount of bait consumed by the hornets. They recorded the approximate number of hornets present on the day of treatment (Day 0) and two (day +2), seven (day +7) and fourteen (day +14) days post-baiting (p.b.). Multiple hornet counts were conducted in front of the hives and at the bait site to obtain an average count. Moreover, the beekepers made efforts to count hornets consistently at the same hour each time. Beekeepers also had to note if other insect species came to the bait, and they should also evaluate the bees’ foraging activity each day during the trial. The completed questionnaires were sent to the GBA headquarters. Occasionally, beekeepers requested additional bait, because of a new increase of hornets in their apiaries.

To compare the reduction effect (RE) expressed as a percentage (RE%) of hornets on days +2, +7, and +14 p.b., with respect to the count on Day 0, the following formula was used:

RE% = [(number of hornets on Day 0 − number of hornets on day +X p.b.)/number of hornets on Day 0 *×* 100]

where day +X p.b. is the day of counting (+2, +7 or +14 p.b.). When the count on day +X was higher than that on Day 0, the RE was considered 0.

### 2.6. Statistical Analyses

All statistical analyses were performed using the statistical software R version 3.6.2 [51]. The differences between the number of hornets on the day of treatment and the bait consumed in each apiary were evaluated using the Kruskal–Wallis rank sum test. The decrease in hornet counts was studied using generalized linear mixed models (GLMMs), where the dependent variable was a quantitative variable (hornet counts), and fixed (day after baiting) and random (apiary) effects were incorporated. GLMMs were analyzed using the ‘lme4′ package with Poisson distribution (log-link function). The best model was selected using the ‘dredge’ function from the ‘MuMIn’ package of R software, which generates, given a full model, a subset of models and selects the model that best fits the data, based on Akaike Information Criterion corrected to sample size (AICc). The overall fit of the best model was assessed by residual analysis and comparison with the null model (with an intercept and random effects only) using the likelihood ratio test. Models were created for each group of apiaries (<10 hornets, between 10–30 and >30 hornets). The correlation between the number of hornets on Day 0 and the bait consumed, and the correlation between the reduction of hornets on days +2, +7 and +14 p.b. and the consumption of bait on Day 0 were analyzed using Spearman’s rank correlation test.

## 3. Results

### 3.1. Larval Inactivation Assays in the Laboratory

The number of larvae fed different concentrations of fipronil and the control group of larvae included are summarized in Table 1. Comparing the effects of toxic baits in the different groups of larvae, those fed with the bait containing 0.01% fipronil presented a higher percentage of affected larvae at 24 h (Table 1).

A 100% mortality was observed 48 h after the administration of the toxic baits. The control larvae did not show any change in their activity and reacted to touching and moving their jaws in response to food requests. After 48 h, control larvae were still alive.

**Figure 1:**
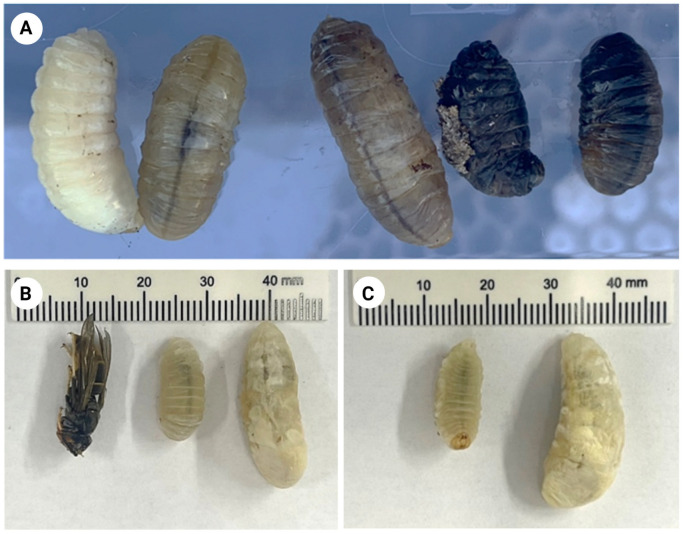
Color changes observed in the *Vespa velutina* larvae (**A**), and aspect of swollen larva in comparison with control larva (**B**,**C**) within 48 h after feeding with fipronil.

Most of the larvae fed with protein baits at different concentrations of fipronil showed a progressive change in color, turning to a blackish color when they died (Figure 1). To determine whether the change in color was due to fipronil ingestion, the control larvae were kept without food until they died. Ten days after the beginning of the experiment, the control larvae lost activity until they died, turning their whitish color to black, as occurred with the larvae fed fipronil.

A swelling effect was also observed in the larvae fed baits containing different concentrations of fipronil (Figure 1). However, this change was not observed in control larvae.

### 3.2. Analysis of the Fipronil Content in Dead Larvae

The HPLC-DAD method, developed and validated using the SANTE guide, was applied to the determination of fipronil content in dead larvae and bait. The quality control of the protein baits used for the efficacy study containing 0.01% fipronil was satisfactory, obtaining a biocide content of 0.0102% ± 0.0001 (*w*/*w*). Each larva was fed a dose of protein bait containing 0.01% biocide equivalent to 1.16 × 10^−3^ mg of fipronil.

The concentration of fipronil found in dead larvae, expressed as mg of fipronil per larva, varied from 1.49 × 10^−4^ mg to 3.47 × 10^−4^ mg.

### 3.3. Testing the Efficacy of Protein Baits with Fipronil in Apiaries

Once hornet activity was confirmed, field trials started. The total number of beekeepers participating in the field trials was similar in 2019 and 2020 but lower in 2021 (Table 2) because this year’s beekeepers reported a lower intensity of *V. velutina* in the apiaries, and the GBA insisted that baiting, to be effective, should be carried out when the presence of hornets in the apiaries was at least moderate (>20); thus, the number of beekeeper participants decreased in 2021. This difference was reflected in the number of apiaries and hives treated, in the number of baits carried out and in the amount of bait delivered by the GBA each year (Table 2). Although beekeepers were urged to complete the questionnaire with information on the bait consumed and the counting of hornets after baiting, only 40.5% (90/222) of the beekeepers submitted it. A total of seven questionnaires were completed in August, 62 in September and 21 in October.

**Figure 2:**
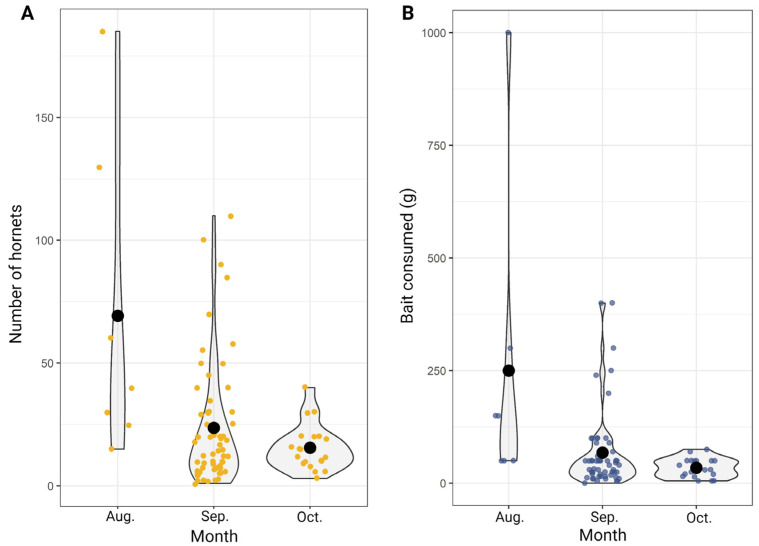
Counts of hornets in front of the hives on the day of treatment (**A**) and the amount of bait consumed in each apiary (**B**) over the three months in which the trials were carried out. The black dots represent the mean. Orange and blue dots represent individual observations.

Considering the results compiled in the 90 questionnaires, an average of 22.6 hornets were present in the apiaries on the day of the start of the baiting (range 1–186). Both the average number of hornets and bait consumption (Figure 2) were significantly higher in August than in September and October (*p* = 0.01204 and *p* = 0.0147, respectively), which did not show significant differences.

The large variability in the number of hornets present in the apiaries had a great influence on the amount of bait transported to the nests and on the RE% observed in the days following baiting (Table 3). An overall reduction of hornets of 40% was observed, but this value was significantly lower (RE% ≈ 25) in the group of apiaries with a low pressure of hornets (<10 hornets) and much higher (RE% ≈ 75) in the group with a higher presence of hornets in the apiary (>30 hornets).

**Figure 3:**
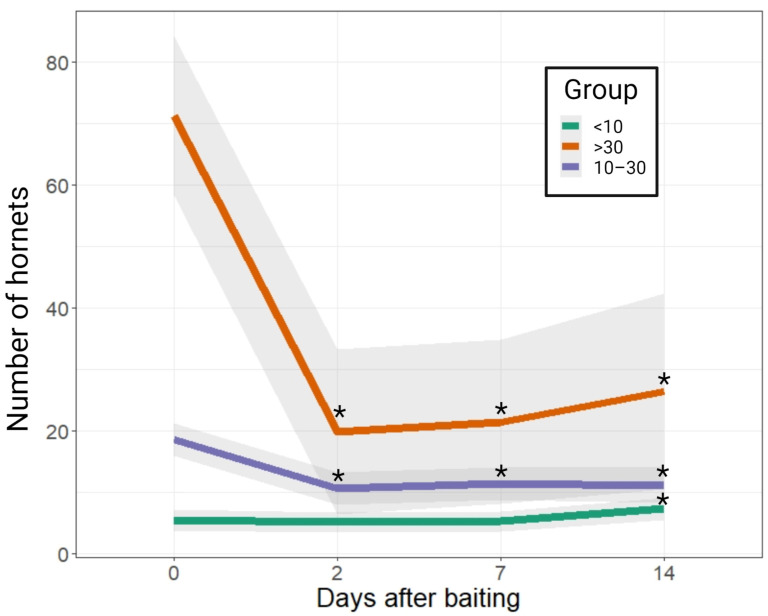
Overall mean evolution of the number of hornets during the field trial [*, *p* < 0.05].

The decrease in the number of hornets observed in the group of apiaries with a higher pressure of *V. velutina*, in addition to being more pronounced 48 h p.b., remained constant throughout the next two weeks (Figure 3). Post-baiting counts on days +2, +7 and +14 were significantly lower than on Day 0 for the two groups of apiaries with a higher number of hornets (Table 4; Figure 3). In the group with a lower pressure of *V. velutina*, although there was a slight reduction in the number of hornets on day +2 and day +7, the decrease was not significant (Table 4; Figure 3).

**Figure 4:**
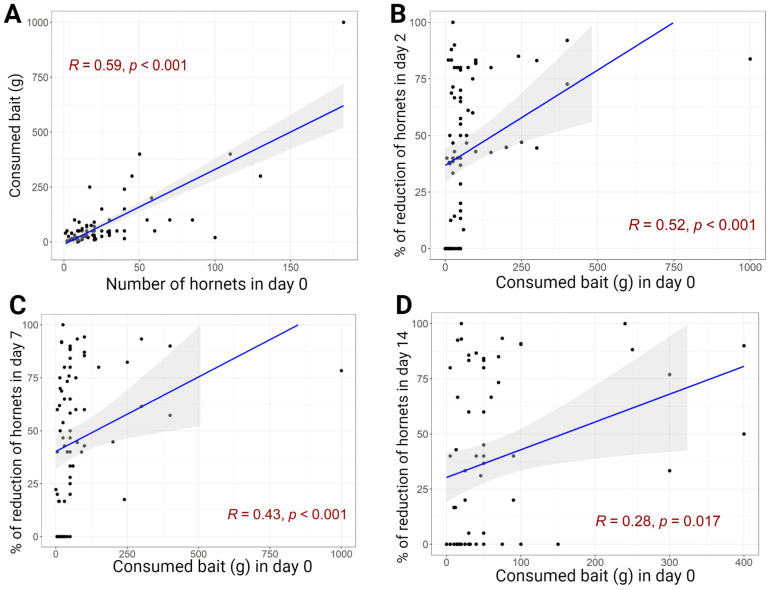
Correlation between the number of hornets in the apiaries and the bait consumed on Day 0 (**A**). Correlation between the bait consumption on Day 0 and the percentage of reduction (RE%) on days +2 (**B**), +7 (**C**) and +14 (**D**) post-baiting; R = Spearman’s rank correlation rho. The shaded area corresponds to the 95% confidence interval of the regression line (in blue).

A significantly positive correlation was observed between the number of hornets present on the day of baiting and the amount of bait transported (Figure 4). In addition, a significant correlation between the amount of bait transported and the observed decrease in hornet counts on days +2, +7 and +14 p.b. was observed (Figure 4).

Regarding other insects that may have been attracted to the toxic baits, the beekeepers reported the presence of some flies, which left quickly when they suffered predation from *V. velutina*. Occasionally, the presence of some European hornet (*Vespa crabro*) was also reported, although it generally left the apiary when several specimens of *V. velutina* appeared. The occasional presence of social wasps, which were immediately preyed upon by Asian hornets, was also reported.

## 4. Discussion

Nest removal and trapping founder queens in spring and workers in summer are the most common techniques used for *V. velutina* control [27,28,29,52]. The best strategy is to combine different methods throughout the annual activity period of the hornets. In this study, we present another method successfully used with other members of the Vespidae family [38,40,41,47] based on chemical control with toxic baiting. Several biocides have been investigated to control wasps, and fipronil provided optimal efficacy at low doses [53,54]. In fact, a protein bait with fipronil has recently been commercialized in New Zealand for this purpose [41]. First, it is essential that the bait is palatable and does not cause any kind of rejection by hornets. In this respect, over the three years, no beekeepers indicated in the questionnaires that they had observed any rejection of the bait by hornets. Second, a low dose of biocide in the bait is key because adult hornets need to survive bait exposure and be able to transport the bait to the nest to feed the larvae. In the current study, the in vivo assays feeding larvae with baits containing different concentrations of biocide showed that the inactivation percentage of larvae increased with the concentration, obtaining 84.1% inactivation after 24 h for the maximum concentration of fipronil tested (0.01%). The fipronil remains found in dead larvae represented 13% to 30% of the total biocide administered. This low concentration could be attributed to the metabolism and excretion processes of the biocide. Despite this low fipronil content in the larvae, it is remarkable to consider that the major metabolites of fipronil (fipronil sulfone, fipronil desulfinyl) [55,56,57] have a higher toxicity than the biocide itself, assuring the efficacy of these protein baits. In addition, the most important effect of feeding larvae with toxic baits in laboratory conditions was the swelling experimented by larvae, which was not observed in the control group of larvae.

Thus, the protein bait with 0.01% fipronil was selected for the efficacy study in the apiaries, showing a significant effect in reducing the presence of *V. velutina*. It was observed that to minimize the pressure of *V. velutina* in the apiary, the amount of toxic bait transported to the nests must be high. However, bait consumption depended on the number of hornets present in the apiary with optimal results when more than 30 hornets were present at the same time in front of the hives. The reduction in the number of hornets in the group of apiaries with >30 hornets (≈75%) was similar to other studies that used protein baits with fipronil to control *Vespula* species in natural environments [39,47,58], but lower in comparison to others [38,41]. However, the concentration of fipronil used in the present study is ten times lower (0.01% *w*/*w*) than the concentrations of fipronil used to control other species of Vespidae (0.1% *w*/*w*) [59]. In addition, we are not aware of other publications about the efficacy of toxic protein baits to control *V. velutina* in apiaries, and unfortunately, we cannot compare the present results with other studies performed in similar emplacements. Moreover, according to the observations of the beekeepers, the reduction achieved in the apiaries with a high predation allowed the honey bees to continue their normal activity.

The duration of the RE% was not affected by the number of hornets on Day 0, and the effect was maintained in a similar way in the three groups of apiaries over the two weeks of the experiments. Although hornet counting did not continue after +14 post-baiting days, some beekeepers reported an increase in hornets but did not reach the initial count. These increases were probably due to the hatching of new hornets that were in the pupal stage at the time of baiting and consequently were not fed with the toxic bait. Additionally, after the initial decline, a rapid increase in hornets was also reported in one apiary. This may be due to hornets coming from other nests because of the lower competition between hornets after baiting compared to the previous days. In the group of apiaries with a low hornet count (<10), a few apiaries showed a slight decrease in the number of hornets but quickly recovered it.

Several authors found that increasing the intensity of baitings increases their efficacy [38,47,58]. As seen in this study, the use of biocides such as fipronil can be useful to control *V. velutina* populations in the periods of highest pressure in the apiaries. However, it is worth noting that degradation residues of fipronil can have acute and chronic effects on bees, affecting the survival and development of bee colonies, and other nontarget species [60,61]. Therefore, it would be necessary to minimize the impact that dead larvae and adult hornets might have, together with the part of the transported bait that does not reach the nests, on other insects, birds, and the environment. Therefore, the use of protein baits with biocides should be as restricted as possible, optimizing the conditions for maximum consumption in the shortest possible time. These conditions can be met if a highly palatable bait is available, and beekeepers use this strategy when apiaries are heavily predated by *V. velutina* at the end of summer and beginning of autumn, when *V. velutina* nests grow rapidly, under the control and supervision of local authorities.

Control of *V. velutina* in apiaries not only benefits beekeeping but also preserves the local entomological fauna, which is also a target of the *yellow-*legged** hornet. In this regard, studies carried out in France indicate that several species of social wasps may account for a third of the prey of *V. velutina* [62].

## 5. Conclusions

The demonstration that a protein bait with a 0.01% concentration of fipronil was effective in inactivating and killing the larvae made it possible to use very small amounts of this biocide in the field trials carried out in apiaries. The use of protein baits with fipronil in apiaries when the presence of *V. velutina* workers is high and can reduce the pressure of the hornets on the hives for at least two weeks after baiting. Furthermore, bees can recover their normal activity. If this type of protein bait with biocide is used in apiaries when the males and gynes are in the larval stage, it could be an appropriate strategy to significantly reduce and control the population of this invasive species and reduce its impact on the local entomofauna. Moreover, it is necessary to study the risks that dead larvae and hornets and the remains of bait may represent for birds, other insects and the environment.

## Figures and Tables

**Table 1:** Number of *Vespa velutina* larvae fed baits containing different concentrations of fipronil, number of control larvae, and number of inactive larvae at 24 h postfeeding.

Fipronil % (*w*/*w*)	Fed Larvae	Inactive Larvae (%) at 24 h
0.003	26	42.3
0.004	33	63.6
0.007	48	68.8
0.01	44	84.1
Control	46	0

**Table 2:** Number of beekeepers that participated in the field assays, number of apiaries and hives treated, quantity of bait distributed, and number of questionnaires completed by beekeepers over the three years.

Year	No. Beekeepers	No. Apiaries	No. Hives	No. Baitings	Bait (kg)	Questionaries Fulfilled
2019	96	153	1562	169	23.4	34
2020	97	117	1004	123	14.2	37
2021	29	31	290	36	7.2	19

**Table 3:** Mean evolution of the number of hornets (mean [Ⴟ] and standard deviation [SD]) and overall reduction effect [RE%] along the field trials; Mean and RE% values after grouping the apiaries according to the number of hornets present on Day 0.

Group of Apiaries		Day 0	Day +2	Day +7	Day +14
All apiaries	Ⴟ (SD)	22.6 (24.5)	10.2 (9.2)	11.1 (13.1)	10.7 (9.7)
	RE%		41.8	43.4	38.8
Apiaries <10 hornets	Ⴟ (SD)	6.5 (2.7)	5.6 (3.9)	5.2 (3.1)	7.1 (6.5)
	RE%		26.4	32.4	25.1
Apiaries 10–30 hornets	Ⴟ (SD)	20.9 (6.4)	13.1 (10.4)	14.1 (13.0)	13.7 (10.3)
	RE%		45.4	43.8	41.7
Apiaries >30 hornets	Ⴟ (SD)	67.1 (28.9)	15.6 (10.7)	19.2 (21.2)	14.9 (13.7)
	RE%		74.0	70.1	77.4

**Table 4:** Summary of GLMMs by categories of apiaries according to the pressure of *Vespa velutina* [***, *p* < 0.001; *, *p* < 0.05; NS, nonsignificant].

Group of Apiaries		Estimate	Std. Error	z-Value	*p*-Value
Apiaries <10 hornets	Intercept	1.55763	0.13537	11.506	<0.001 ***
	Day +2	−0.06645	0.12064	−0.551	0.582 ^NS^
	Day +7	−0.03637	0.11971	−0.304	0.761 ^NS^
	Day +14	0.26343	0.11328	2.325	0.020 *
Apiaries 10–30 hornets	Intercept	2.86838	0.09516	30.144	<0.001 ***
	Day +2	−0.56898	0.05706	−9.971	<0.001 ***
	Day +7	−0.50479	0.05626	−8.972	<0.001 ***
	Day +14	−0.49360	0.05901	−8.365	<0.001 ***
Apiaries >30 hornets	Intercept	3.99687	0.10193	39.21	<0.001 ***
	Day +2	−1.27011	0.06120	−20.75	<0.001 ***
	Day +7	−1.16684	0.05938	−19.65	<0.001 ***
	Day +14	−0.86979	0.06366	−13.66	<0.001 ***

## Data Availability

The data presented in this study are available on request from the corresponding author.
